# WRIST ARTHROSCOPY: BASIC TIPS FOR DRY ARTHROSCOPIC EXPLORATION

**DOI:** 10.1590/1413-785220172506160670

**Published:** 2017

**Authors:** HENRIQUE DE BARROS PINTO, SUZILAINE RAMOS DE OLIVEIRA, FLAVIA CURVO PEREIRA, NILTON MAZZER

**Affiliations:** 1. Hospital Federal da Lagoa, Rio de Janeiro, RJ, Brazil.; 2. Faculdade de Medicina de Ribeirão Preto da Universidade de São Paulo, SP, Brazil.

**Keywords:** Wrist/pathology. Arthroscopy/methods, Compartment syndromes, Learning curve, Treatment outcome, Punho/patologia, Artroscopia/métodos, Síndromes compartimentais, Curva de aprendizado, Resultado do tratamento.

## Abstract

**Objective:**

This article provides details and tips on the dry arthroscopic technique, based on our experience and its clinical applications.

**Method:**

The technique was applied to 65 patients (33 men and 32 women) aged between 20 and 62 years (average of 35.4 years) for treating: synovial cyst resection, scapholunate ligament injury repair, ulnocarpal impact correction, triangular fibrocartilage injury repair, and assisted reduction of distal radius fractures.

**Results:**

A minimally invasive intra-articular evaluation has been observed as a benefit, with low infection rate, small scars, and high rates of early recovery, without affecting intra-articular fluid use, reducing the risk of compartment syndrome and infiltrated soft tissues, in the case of need for associated open surgery. As for the difficulties, we report the surgeon’s view, which is commonly prevented by optical blurring or debris that hit the lens, and the need for radiofrequency care, since the heat generated is dissipated with greater difficulty than in the classical technique.

**Conclusion:**

Dry arthroscopy emerges as an effective choice to treat wrist pathologies, however, deep knowledge and ease with the classical technique, as well as a learning curve, are key to obtain a good outcome. **Level of Evidence V, Expert Opinion.**

## RESUMO

**Objetivo:**

Este artigo apresenta detalhes e dicas sobre a técnica de artroscopia seca, baseada em nossa experiência e em suas aplicações clínicas.

**Método:**

A técnica foi aplicada em 65 pacientes (33 homens e 32 mulheres) com idades entre 20 e 62 anos (média de 35,4 anos) para o tratamento de ressecção de cisto sinovial, reparo de lesão do ligamento escafo-semilunar, correção do impacto ulnocarpal, reparo de lesão da fibrocartilagem triangular e assistência na redução de fraturas da parte distal do rádio.

**Resultados:**

A avaliação intra-articular minimamente invasiva foi observada como benefício, com baixo índice de infecção, cicatrizes pequenas e altas taxas de recuperação precoce, sem prejuízo do uso intra-articular de líquido, reduzindo o risco de síndrome compartimental e tecidos moles infiltrados, no caso de necessidade de cirurgia aberta associada. Quanto às dificuldades, relatamos a visibilidade para o cirurgião, comumente impedida pelo turvação da óptica ou detritos salpicados na lente e a necessidade de cautela com a radiofrequência, pois o calor gerado é dissipado com maior dificuldade do que na técnica clássica.

**Conclusão:**

A artroscopia seca surge como opção efetiva no tratamento das patologias de punho, entretanto, o conhecimento profundo e as facilidades com a técnica clássica, bem como a curva de aprendizado, são fundamentais para obter um bom resultado. **Nível de Evidência V, Opinião do Especialista.**

## INTRODUCTION

Since the 1980s, the use of wrist arthroscopy as a diagnostic tool for intra-articular wrist pathology, which allows minimally invasive treatment of many diseases, has been widely disseminated among surgeons as a routine.

Traditional arthroscopy, or ‘wet’ arthroscopy, uses fluid to distend and create a working cavity. However, distending the joint with fluid is not a complication-free procedure. The fluid infiltrates the tissues, escapes through the gateways, and this can cause serious problems, such as the compartment syndrome. Finally, the use of fluid greatly complicates any concomitant surgery after arthroscopic exploration, making it difficult to combine arthroscopy with open procedures, such as osteotomies and ligament reinsertions, e.g. for triangular fibrocartilage, due to the loss of anatomical frame definition by the massive fluid infiltration.^1^ ^,^ ^2^

This article is a retrospective study based on surgical experience by means of 65 wrist arthroscopies, using the dry technique, whose aim is providing the reader with a description of the surgical technique, its challenges, and tips, allowing its reproduction.

## MATERIAL AND METHODS 

Between April 2013 and September 2015, 65 patients underwent an arthroscopic wrist procedure using the dry technique for treating pathologies and defining the diagnosis. These medical records were analyzed within the period from October 2015 to December 2015; 32 patients were women and 33 were men; their ages ranged from 20 to 62 years (average of 35.4 years); 40 patients had synovial cysts; 16, triangular fibrocartilage injury; 5, distal radius fracture; 5, scapholunate ligament injury; and 2, ulnocarpal impact. Because this is an analysis of medical records belonging to previously operated patients, this study did not require the use of a free and informed consent term and it has not been submitted to the evaluation of a research ethics committee.

**Figure 1:**
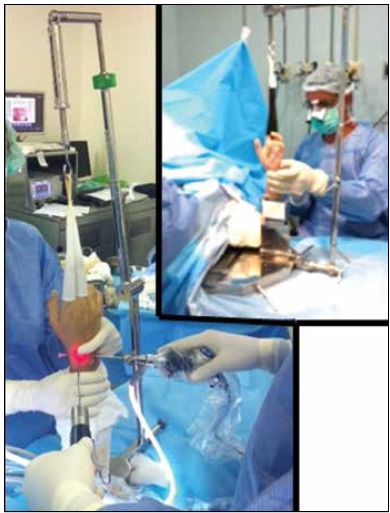
Positioning in the traction tower.

The surgical technique was applied with the patient in the supine position on the operating table, under regional anesthesia - axillary block and sedation, with the shoulder along the table edge. The shoulder was abducted, and the forearm, vertically suspended, using Chinese mesh on the index and middle fingers by a traction system supported by the surgical table. The traction obtained was measured by a dynamometer, obtaining values ​​between 5 and 8 kgf. After exsanguination, the pneumatic cuff above the elbow was inflated between 250-300 mmHg. (Figure 1) The arthroscope used was 2.5 mm with a viewing angle of 30º. There were basically two gateways, 3-4 and 6R, which depending on the pathology, work either as a visualization gateway or as an instrumentation gateway. Radial pathologies use the gateway 6R for visualization, while ulnar pathologies use the gateway 3-4 for this purpose. The gateway 3-4 is established 1 cm distal to Lister’s tubercle, with a 40 × 12 mm needle inserted first at a 10° volar angle and parallel to the articular surface, thus reducing the risk of cartilage damage. The gateway 6R is established by carpal ulnar extensor tendon palpation, and the fixed radial point to the tendon palpated is then also demarcated using a needle. When the viewing portal is determined, the demarcation needle is removed and a longitudinal incision is obtained with a number 15 scalpel blade, dilated with hemostatic forceps, then a trocar is inserted into the joint for optic input.

All the arthroscopic explorations were performed using the dry technique, allowing the identification of major points to be addressed. So, we observe the following aspects: 

Regarding the determination of gateways, once the gateway for visualization has been chosen, the needle demarcating the instrumentation gateway must be kept to ensure, by internal vision, its proper positioning, posterior incision, dilation, and introduction of instruments.

**Figure 2:**
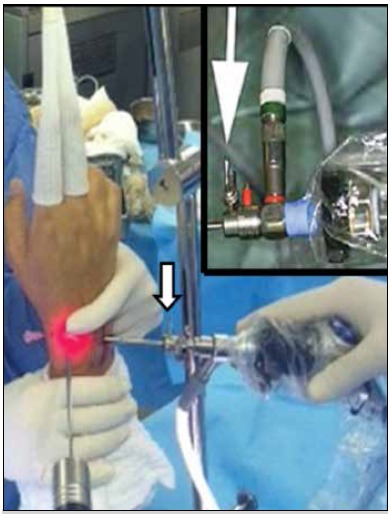
Scope valve kept open.

Scope valves should be kept open throughout the procedure to allow air to circulate freely in the joint cavity. Otherwise, the shaver suction of the does not work properly, causing collapse and preventing full visualization of the space. (Figure 2)

One of the scope valves should be pre-prepared with a 20 mL syringe of 0.9% saline, positioned in the scope to promptly wash the joint cavity, functioning as a pre-assembled irrigation system, used as needed and still not de-characterizing the dry technique.

Salt irrigation is sometimes needed to clean the field or cool the shaver. The joint should be irrigated to remove debris and blood whenever needed. Optical blurring, as well as the presence of blood and spatter, can obscure the surgeon’s view. And continued shaver use can generate its heating as a result of friction, perceptible in the surgeon’s hand, with possible device damage.

Regarding the possibility of thermal damage to the cartilage and adjacent soft tissues, caused by the use of radiofrequency, it is recommended to use them only briefly and punctually, and never continuously, also using the irrigation system whenever needed.

When used simultaneously with osteosynthesis of the intra-articular fracture of the distal radius, the gateway 4-5 and the knee probe help achieving and controlling joint surface reduction.

During the triangular fibrocartilage reinsertion, the dry technique has the advantage of no liquid resistance, which occurs with the classical technique.

## DISCUSSION

**Figure 3:**
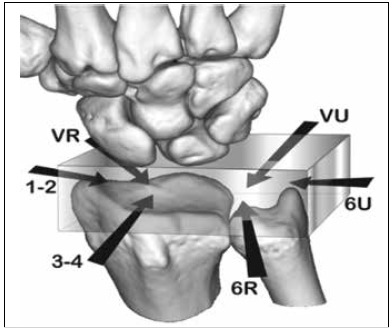
Box concept, the wrist can be observed in many directions.^5^

Arthroscopic wrist examination should include the radiocarpal and mediocarpic joints. Several gateways are described, each having a technique of its own and adequate function. Traditionally, 3-4, 4-5, 6R and midcarpal gateways have been used as visualization and working gateways. The traditional gateways for wrist arthroscopy are dorsal because of the presence of fewer neurovascular structures in the wrist dorsum, as well as the initial emphasis on assessing their volar ligaments.^3^ ^,^ ^4^ With the advent of new volar gateways, it is possible to have visualization and working gateways that surround the wrist as a whole.^5^ ^,^ ^6^ This allows the surgeon to use the arthroscope for display and instrumentation in all directions - the box concept. (Figure 3)

These are some of the indications for wrist arthroscopy: it is a useful tool for diagnosis in patients with wrist pain, limited arc of movement, and reduced force, in which a non-invasive diagnosis and a conservative treatment have failed. And also in synovial cyst resection, especially in cases where the patient has concomitant wrist pain, distal radius fractures with deviation greater than 2 mm, isolated radial styloid fractures, distal radius fractures with suspected associated ligament or capsular injury, Reparable peripheral injuries of triangular fibrocartilage, among others, for which minimally invasive solutions are sought.^7^

As for contraindications of classical arthroscopy, some typical ones are the large capsular injuries, which have fluid leakage risk,^8^ active infection, neurovascular impairment, and distorted anatomy. In addition to these, there are distal radius fractures with metaphyseal comminution and shear and volar fractures, as they require open treatment, although the arthroscope can be inserted to help reducing the joint. Compartment syndrome risk has also been considered a contraindication for arthroscopy, particularly after severe fractures.

Rupenian reports that performing traditional arthroscopy, introducing fluid in an attempt to maintain the optic cavity, is not a problem-free procedure.^9^ The imbalance between fluid input and output associated with extravasation through gateways often results In loss of visualization and risk of compartment syndrome. We observed that dry arthroscopy sets aside all concerns about intra-articular fluid management and pressure, and thanks to the lack of tissue infiltration, the wrist volume and contour are preserved during surgery.

In other surgery fields, such as laparoscopy, water is not used to keep the optic cavity; instead, gas is used. Gas (air, carbon dioxide, oxygen, or nitrous oxide) was the first intra-articular substance used to distend the joint and perform arthroscopy since the first description of this procedure by Bircher, in 1921.^10^ Levin et al.^11^ devised a balloon for this purpose that, when placed between the soft tissue frames, creates air pockets that serve as actual optical cavities, facilitating the dissection of free flaps. Friedlander and Sundin^12^ used external skin traction, creating a cavity without fluid insufflation, to facilitate minimally invasive dissection of the dorsal large muscle flap. These researchers used soft tissue traction to develop the optic cavity when they took up the flaps.^11^ ^,^ ^12^ To summarize, water is neither crucial or needed to determine any cavity. It was then considered that in the wrist, traction itself would keep the optic cavity open.^13^

Another major advantage of the dry technique reported by Rupenian^9^ is the possibility of assessing injuries in their natural setting. We notice, for instance, in the excision of cysts and pathologies involving the presence of synovitis, that the infiltration of tissues by the fluid prevents a rather real view of anatomical structures, which are distorted and distended; however, in the dry technique these structures show a higher definition.

Slutsky^3^ reported the benefit of providing reduced intra-articular distal radius fractures with arthroscopic assistance through the dry technique by eliminating the concern with fluid extravasation. We observed that an advantage of the dry technique is being able to perform an open procedure, such as volar plate osteosynthesis of the distal radius, concomitant with arthroscopy.

Del Piñal et al.^13^ and Del Piñal^1^ mention in their studies some details and tips to put the dry arthroscopic technique into practice and to guarantee a safe and uneventful procedure: they indicate that, sometimes, the surgeon’s view may not be so clear due to the presence of blood and debris, generating optical blurring, which can overshadow the optic tip. The researchers have used a neurosurgical swab for cleaning the field, whose need was discarded over time. The currently used method is a pre-assembled irrigation system that has a syringe installed in the scope valve which, when needed, makes it possible to use small amounts of fluid in order to clean the field. With a refined operative technique, we observed that, during the procedures, the amount of fluid required was gradually lower.

According to Del Piñal,^1^ the suction needed to clean the field paradoxically blurs the vision by agitating the joint contents and generating the cavity collapse. So, the researcher advises to open the shaver suction only when aspiration is needed. We kept the scope valve open at all times for free air circulation in the cavity and the suction used only when needed.

One of the surgeons’ concerns in terms of dry arthroscopy is the possibility of thermal damage within the joint by the use of radiofrequency. Del Piñal^1^ reports that the heat generated may have difficulty to dissipate, and there is a risk of thermal damage to soft tissues and cartilage surfaces. For this reason, the author does not advise to use this type of device continuously, without fluid to cool. In case of need, we do not see any problem to use the irrigation system, but this does not characterize the dry technique.

Del Piñal^1^ ^,^ ^14^ reported intercurrence cases observed with regard to the shaver heating during its use, perceptible to the surgeon’s touch, preventing her/him from continuing the procedure without proper cooling. The rotation mechanism of these instruments heats up, as a result of friction, when used for long periods of time. Such heating, obtained as an intercurrence in three of our procedures, is easily overcome when we irrigate the device externally with saline, which cools it and makes it fit to be used again.

Still according to Del Piñal et al.,^13^ the dry technique has a learning curve not only to overcome the difficulties secondary to vision and overshadow, but because some signs and findings differ from those observed in the classical technique. These differences between the two techniques do not prevent a surgeon familiar with the classical arthroscopy technique from rapidly incorporating the dry technique and benefiting from its advantages. Just as it occurs with any change from a familiar technique to a new one, there is a need to be prepared to accept some frustration at first. We adopted progressive changes until developing and adapting to this operative procedure, making good use of it and obtaining results considered satisfactory.

Del Piñal et al.,^13^ in his works, does not mention the technique used to work up the mediocarpic joint, either the dry or classic technique. In our experience, we performed the mediocarpic joint arthroscopies using irrigation with saline solution, through a syringe adapted to scope.

## CONCLUSION

Wrist arthroscopy with the dry technique has shown to be a safe procedure to detect and treat wrist pathologies. However, it is understood that this procedure requires a systematic approach, knowledge on the technique, and a learning curve to minimize complications and ensure successful outcomes.

## References

[1] Del Piñal F (2011). Dry arthroscopy and its applications. Hand Clin.

[2] Del Piñal F (2011). Technical tips for (dry) arthroscopic reduction and internal fixation of distal radius fractures. J Hand Surg Am.

[3] Slutsky D, Slutsky DJ, Nagle DJ (2007). Wrist arthroscopy portals. Techniques in hand and wrist arthroscopy.

[4] Berger RA (1999). Arthroscopic anatomy of the wrist and distal radioulnar joint. Hand Clin.

[5] Bain GI, Munt J, Turner PC (2008). New advances in wrist arthroscopy. Arthroscopy.

[6] Van Meir N, Degreef I, De Smet L (2011). The volar portal in wrist arthroscopy. Acta Orthop Belg.

[7] Slutsky D, Trumble T (2007). Wrist arthroscopy: Portals and procedures. Hand surgery update IV.

[8] Slutsky DJ, Nagle DJ (2008). Wrist arthroscopy current concepts. J Hand Surg Am.

[9] Rupenian P (2013). Dry arthroscopy of the shoulder. Arthrosc Tech.

[10] Bircher E (1921). Die Arthroendoskopie. Zentralbl Chir.

[11] Levin LS, Rehnke R, Eubanks S (1995). Endoscopic surgery of the upper extremity Hand. Clin.

[12] Friedlander L, Sundin J (1994). Minimally invasive harvesting of the latissimus dorsi. Plast Reconstr Surg.

[13] Del Piñal F, García-Bernal FJ, Pisani D, Regalado J, Ayala H, Studer A (2007). Dry arthroscopy of the wrist surgical technique. J Hand Surg Am.

[14] Del Pinal F (2008). Dry arthroscopy of the wrist its role in the management of articular distal radius fractures. Scand J Surg.

